# External validation of the DAYS score for suspected deep vein thrombosis

**DOI:** 10.1016/j.rpth.2025.102885

**Published:** 2025-05-22

**Authors:** Thor–David Halstensen, Camilla Hardeland, Waleed Ghanima, Vigdis Abrahamsen Grøndahl, Aliaksandr Hubin, Camilla Tøvik Jørgensen, Kerstin de Wit, Mazdak Tavoly

**Affiliations:** 1 Faculty of Health, Welfare and Organisation, Department of Nursing, Health and Laboratory Science, Østfold University College, Fredrikstad, Norway; 2 Institute of Clinical Medicine, University of Oslo, Oslo, Norway; 3 Internal Medicine Clinic, Østfold Hospital Trust, Sarpsborg, Norway; 4 Department of Hematology, Oslo University Hospital and Institute of Clinical Medicine, University of Oslo, Oslo, Norway; 5 Østfold University College, Fredrikstad, Norway; 6 Department of Mathematics, University of Oslo, Oslo, Norway; 7 Department of Emergency Medicine, Østfold Hospital Trust, Sarpsborg, Norway; 8 Department of Emergency Medicine, Queen’s University, Kingston, Ontario, Canada; 9 Department of Medicine, McMaster University, Hamilton, Ontario, Canada; 10 Department of Health Research Methods, Evidence, and Impact, McMaster University, Hamilton, Ontario, Canada; 11 Department of Medicine, Sahlgrenska University Hospital, Gothenburg, Sweden

**Keywords:** deep vein thrombosis, decision support systems, clinical, clinical decision rules, risk assessment

## Abstract

**Background:**

Diagnosing deep vein thrombosis (DVT) involves clinical assessment, D-dimer testing, and imaging. The DAYS score, a novel 2-item prediction tool combined with D-dimer, demonstrated promising performance but required external validation.

**Objectives:**

This study aimed to validate 2 DVT prediction scores: the DAYS score and a newly developed DVT score.

**Methods:**

Data were collected from a prospective Norwegian DVT management study (2015-2018; NCT02486445). The DAYS score includes 2 items (DVT most likely diagnosis and calf swelling), while the new score comprises tenderness along the deep veins and previous venous thromboembolism. DVT was considered excluded if no items were present and D-dimer was < 1.0 μg/mL or if ≥1 item was present and D-dimer was < 0.5 μg/mL. The 2-tier Wells score served as the reference. Safety was defined as the number of missed DVT cases divided by the total number of patients classified as having DVT excluded and was set at 2%.

**Results:**

Among 1312 patients (median age, 64 years; IQR, 52-73 years; 55% women), 261 (20.0%) had confirmed DVT. The DAYS score excluded DVT in 455 patients (34.6%), of whom 11 were diagnosed with DVT (failure rate, 2.4 %; 95% CI, 1.2-4.2). The new score excluded DVT in 519 patients (39.6%) and missed 7 cases with confirmed DVT (failure rate, 1.3%; 95% CI, 0.5-2.8). The Wells score excluded DVT in 271 patients (20.6%), missing only 2 cases with confirmed DVT.

**Conclusion:**

While both the DAYS score and the new score demonstrated low failure rates, they exceeded the predefined safety threshold.

## Essentials

• The new DAYS score for deep vein thrombosis (DVT) underwent external validation.
• Data came from a Norwegian DVT study (2015-2018) including 1312 adults with suspected DVT.
• The DAYS score did not meet the safety threshold, as it missed a significant number of DVT cases.
• A novel score missed fewer DVTs, but its potential error range exceeded the goal.

## 1 Introduction

Deep vein thrombosis (DVT) is a frequent acute medical emergency [1]. Current guidelines recommend using a clinical decision rule to standardize management, with the Wells score being considered the gold standard [2,3]. However, while the Wells score in conjunction with D-dimer safely excludes DVT in 30% of patients with suspected DVT [4], the majority require further ultrasound evaluation, with only 20% of patients referred to ultrasound receiving a confirmed DVT diagnosis [5]. Apart from lacking efficiency, the Wells score has by some been regarded as overly cumbersome to use in time-constrained and crowded emergency departments, potentially leading to overriding or miscalculation of the score [[6], [7], [8]]. In addition, it has been argued that an optimal diagnostic decision rule should only include objective categories to enhance standardization [1,9,10].

To address these shortcomings a recent decision rule, the DAYS score, was developed and internally validated using individual patient data from 3368 patients and modeled from the items in the original Wells score [11]. The aim of the study was to create a decision rule with enhanced feasibility (fewer items) and efficiency (reducing the need for ultrasound), albeit retaining adequate safety [11]. However, the score is yet to be externally validated.

Following similar statistical standards as in the DAYS study, our group recently developed and internally validated a novel DVT score containing only objective categories [12]. While our score demonstrated adequate safety compared with the Wells score, it did not improve efficiency because the traditional threshold for D-dimer at 0.5 μg/mL was used [12].

The primary aim of this study was to externally validate the DAYS score in an independent cohort. The secondary aim was to evaluate the safety and efficiency of our score using a higher D-dimer cutoff of 1.0 μg/mL. The performance and accuracy of both scores were compared with those of the Wells score.

## 2 Methods

### 2.1 Study design and participants

This study is a retrospective analysis based on data acquired from the Ri-schedule study (NCT02486445) [13], which was originally designed as a prospective outcome study . The Ri-schedule study encompassed consecutive patients referred to Østfold Hospital Trust’s emergency department in Norway between 2015 and 2018, with suspected DVT. The primary focus of the study was to evaluate the safety of rivaroxaban during the prediagnostic phase of DVT. All participants were followed up for 90 days. In addition, the study collected numerous variables including all items in the original Wells score to enable future refinement of the management of patients with suspected DVT.

### 2.2 Clinical decision rules

The DAYS score algorithm is detailed in the original study and illustrated in Figure [11]. In summary, the algorithm comprises 2 items: DVT assessed as the most likely diagnosis and the presence of calf swelling, defined as a difference of 3 cm in circumference between the affected and unaffected leg. DVT being the most likely diagnosis was defined as the inverse of the alternative diagnoses being more likely in the Wells score. Patients were categorized as having DVT unlikely or likely based on no items present (unlikely) or at least 1 item present (likely). For those deemed unlikely to have DVT, a D-dimer threshold of < 1.0 μg/mL was applied, while the traditional threshold of 0.5 μg/mL was used for patients with at least 1 item present. DVT was considered excluded (without further workup needed) by the DAYS score if the patients had 0 items present and D-dimer was <1.0 μg/mL or at least 1 item was present and D-dimer was < 0.5 μg/mL (Figure). The DAYS score underwent internal validation, meeting the safety benchmark of missing <2% of DVT cases in those deemed not needing further workup [11]. All items in the original Wells score [14] were assessed and registered for every included patient in the Ri-schedule study, thus enabling accurate calculation of the DAYS score.

In our previous study, which involved the development and internal validation of the new DVT score, we identified tenderness along the deep veins and a history of venous thromboembolism (VTE) as the most predictive items for DVT [12]. Notably, our aim was to develop an entirely objective score, thus the subjective category of the Wells score (alternative diagnosis more likely) was omitted from the analyses. The score included 2 categories; DVT unlikely if none of the items were present and DVT likely if 1 or more of the items were present. Patients with 0 items present and D-dimer < 0.5 μg/mL were regarded as having DVT excluded, whereas those categorized as having DVT likely required further evaluation with compression ultrasound (CUS). The score was revealed to be equally safe as the Wells score [12].

The Wells score serves as the reference standard for clinical decision rules to which new scores are compared [1]. Both the DAYS score and the new DVT score are dichotomized as DVT being likely or unlikely based on the number of items present. Consequentially, to facilitate comparison between the 3 scores we opted for including the 2-tier Wells score, which also categorizes the patients as either unlikely or likely to have DVT [15].

### 2.3 D-dimer test and CUS

In the Ri-schedule study, the STA-Liatest D-Di Plus (Stago Diagnostics) D-dimer test was used in all patients. A cutoff of ≥ 0.5 μg/mL fibrinogen equivalent units was considered as a positive test. Patients with positive D-dimer were referred for whole-leg CUS. The deep and saphenous veins were scanned with a linear probe (5-10 MHz). For first DVT, recurrent contralateral DVT, recurrent ipsilateral DVT with documented resorption of thrombus, or recurrent DVT without available images for comparison, the diagnostic criterion was incompressibility of the vein or a grayscale visualization of the thrombus. Recurrent ipsilateral DVT was defined as noncompressibility of, or visualization of, the thrombus in a venous segment not involved from reference CUS [13]. Distal DVT was defined as any thrombus located distal to the popliteal vein.

### 2.4 Outcomes

The primary outcome of the study was to evaluate safety and efficiency of the DAYS compared with that of the Wells score. Safety was deemed adequate if the upper bound of the 95% CI for the overall DVT prevalence among all patients classified as having DVT excluded did not exceed 2.0%. This threshold for safety was used in the original DAYS study and is generally considered as acceptable [3,4,11]. Efficiency was defined as the proportion of patients who had ultrasound deferred according to the DAYS score compared with that of the Wells score.

The secondary outcomes were the safety and efficiency of the new DVT score and the assessment of performance measures of all 3 scores by calculating the sensitivity, specificity, negative predictive value, positive predictive value, and accuracy.

### 2.5 Statistical analyses

All analyses were performed using the statistical software programs STATA 18 (StataCorp) and R Statistical Software (v4.2.1; R Core Team 2021). Continuous variables are presented as means with SDs for normally distributed data or as medians with IQRs for skewed data. Categorical variables are presented as frequencies with percentages.

DVT was considered as excluded by the scores if either no items were present and D-dimer was < 1.0 μg/mL or at least 1 item was present and D-dimer was <0.5 μg/mL. If these criteria were not met, DVT was considered included, needing further workup. Utility was defined as the total number of patients who were categorized as having DVT excluded by the 2 scores, ie, no further workup required.

CIs for the proportions were calculated using the Clopper–Pearson exact method. The accuracy of the scores was calculated as the proportion of correctly classified cases, defined as the sum of true positives and true negatives divided by the total number of cases (true positives + true negatives + false positives + false negatives).

## 3 Results

Between February 2015 and November 2018, 1653 patients were included in the Ri-schedule study. Of these, 341 patients were excluded because of the following reasons: ongoing anticoagulant treatment (*n* = 249), at least 1 missing value in the explanatory variables such as body mass index (*n* = 52), Wells score (*n* = 14), red discoloration (*n* = 10), suspected infection (*n* = 8), D-dimer (*n* = 7), and VTE in first-degree relative (*n* = 1). Thus, the final study population comprised 1312 patients.

Median age was 64 years (IQR, 52-73 years) and 55% were women; 261 (20%) patients were diagnosed with DVT. Patient characteristics and risk factors are summarized in Table 1. DVT was categorized as unlikely in 260 patients (20%) by the DAYS score, whereas 633 patients (48%) had DVT deemed as unlikely by the Wells score (Table 2).

**Table 1:** Patient characteristics (*N* = 1312).

Characteristic	Value
Age (y), median (IQR)	64 (52-73)
Female sex	723 (55)
Body mass index > 30 kg/m^2^	479 (37)
Symptom duration (d), median (IQR)	7 (3-14)
Previous VTE	187 (14)
Active cancer^a^	61 (5)
Paralysis, paresis, or recent plaster immobilization of lower extremity	53 (4)
Bedridden recently	86 (7)
Tenderness along the deep venous system	808 (62)
Entire leg swollen	269 (21)
Calf swelling > 3 cm	342 (26)
Pitting edema confined to the symptomatic leg	577 (44)
Collateral superficial veins present	154 (12)
Recent surgery	104 (8)
Immobilization due to trauma	64 (5)
Immobilization neurologic disease	28 (2)
Travel >4 h	373 (28)
Hormone-replacement therapy	118 (9)
Pregnancy or puerperium	19 (1)
Hormonal contraceptives	35 (3)
D-dimer (mg/L), median (IQR)	0.8 (0.4-1.7)
DVT	261 (20)
Proximal	174 (67)
Distal	87 (33)

Values are *n* (%) unless specified.

DVT, deep vein thrombosis; VTE, venous thromboembolism.

a Active cancer: cancer diagnosed in the previous 6 months.

**Table 2:** Patients categorized as unlikely or likely to have DVT with corresponding proportions of confirmed DVT according to the 3 scores.

Clinical probability score	All patients (*N* = 1312)	Confirmed DVT (*n* = 261)
DAYS score		
DVT unlikely	260 (20)	18 (6.9)
DVT likely	1052 (80)	243 (93)
New score		
DVT unlikely	450 (34)	36 (14)
DVT likely	862 (66)	225 (86)
Wells score^a^		
DVT unlikely	633 (48)	52 (20)
DVT likely	679 (52)	209 (80)

Values are *n* (%).

DVT, deep vein thrombosis.

a Two-tier Wells score.

Figure displays the number of patients categorized as having DVT unlikely or likely according to the 3 scores and the D-dimer thresholds. According to the DAYS score, 201 patients (15%) were categorized as having DVT unlikely with a D-dimer of < 1.0 μg/mL. Of these, 66 patients (33%) had a D-dimer of 0.5 to < 1.0 μg/mL. An additional 254 patients (19%) were categorized as having DVT likely with a D-dimer of < 0.5 μg/mL. Among the 455 (201 + 254) patients (35%) having DVT classified as excluded by the DAYS score, 11 patients received a final diagnosis of DVT, of which 8 were in the unlikely group with D-dimer of 0.5 to < 1.0 μg/mL, resulting in an overall failure rate of 2.4% (95% CI, 1.2-4.2) (Figure A). The missed DVTs were distally located in 7 cases and proximally in 4 cases.

**Figure:**
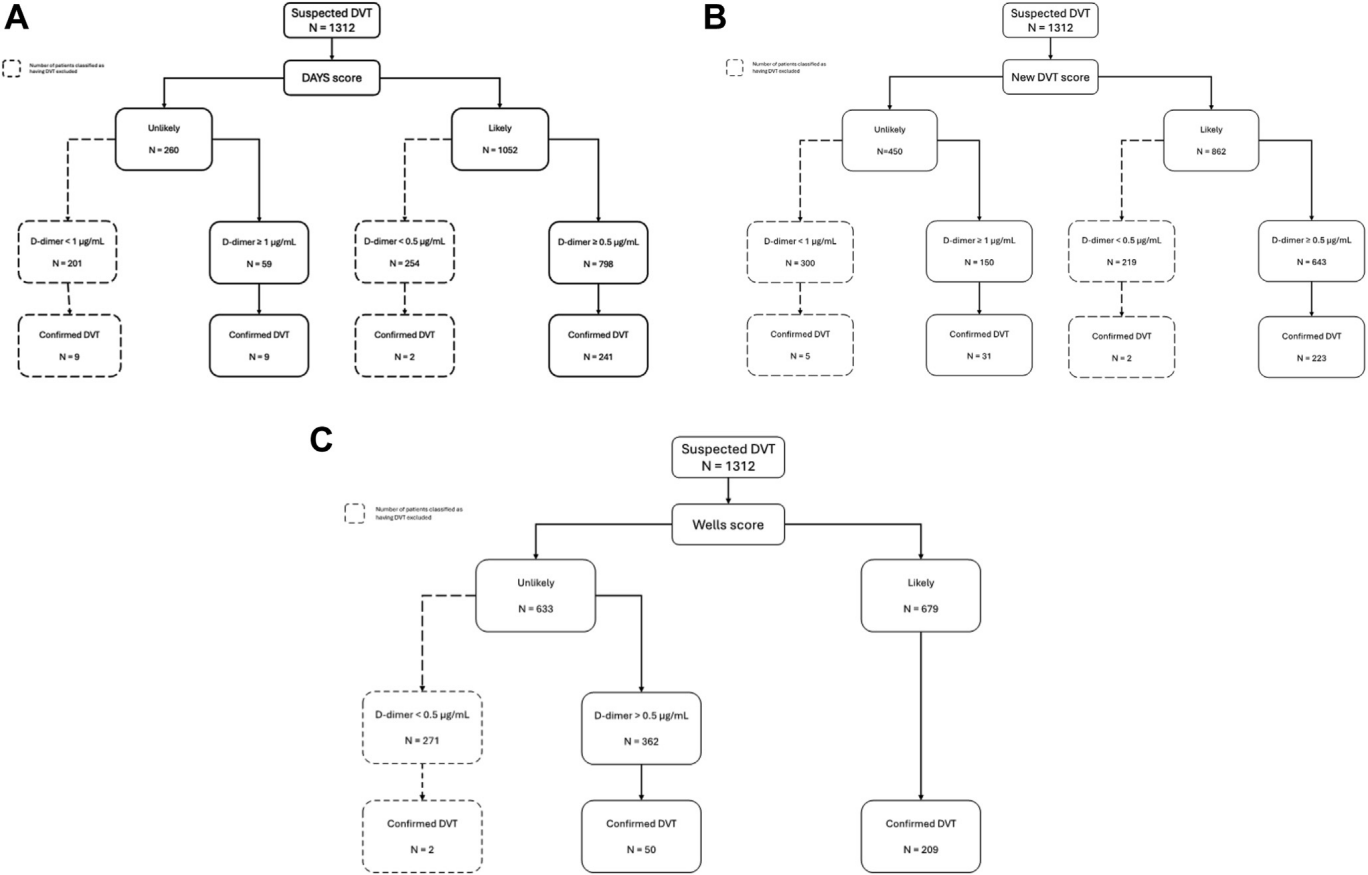
(A–C) Categorization of patients according to the 3 scores and D-dimer. DVT, deep vein thrombosis.

A total of 519 patients (40%) were classified as having DVT excluded according to the new DVT score. Of these, 7 patients were diagnosed with DVT (2 proximal and 5 distal), resulting in a failure rate of 1.3% (95% CI, 0.5-2.8) (Figure B).

The Wells score deemed further workup as unnecessary in 271 patients (21%) (Figure C). In contrast, the DAYS score and the new DVT score identified an additional 184 patients (14%) and 248 (22%), respectively, in whom ultrasound could be withheld.

Table 3 displays the performance of the 3 scores, among which the DAYS score exhibited the lowest sensitivity (95.8%; 95% CI, 92.6-97.9) and negative predictive value (98.2%; 95% CI, 96.9-99.0) (Table 3).

**Table 3:** The diagnostic performance of the 3 scores.

Score performance	DAYS score, value (95% CI)	New score, value (95% CI)	Wells score^a^, value (95% CI)
Sensitivity	95.8 (92.6-97.9)	97.3 (94.6-98.9)	99.2 (97.3-99.9)
Specificity	42.3 (39.2-45.3)	48.7 (45.7-51.8)	25.6 (22.98-28.35)
NPV	98.2 (96.9-99.0)	98.7 (97.2-99.4)	99.3 (97.1-99.8)
PPV	29.2 (28.0-30.4)	32.0 (30.7-33.4)	24.9 (24.2-25.6)
Accuracy	52.9 (50.2-55.6)	58.4 (55.7-61.0)	40.2 (37.6-43.0)

NPV, negative predicative value; PPV, positive predictive value.

a 2-tier Wells score.

## 4 Discussion

This study assessed the external validity of the DAYS score and observed a failure rate of 2.4%, suggesting inadequate safety for clinical implementation. In addition, the new DVT score developed by our group and tested with a D-dimer threshold of 1.0 μg/mL also did not demonstrate sufficient safety when the upper bound of the CI was set at 2.0. However, both scores were able to identify significantly more patients for whom further workup was deemed unnecessary.

The Wells score does not meet current health care requirements of cost-effectiveness and efficiency [16,17]. Consequently, an increasing number of patients are referred for ultrasound, given its noninvasive and radiation-free nature. Similar to recent published decision rule studies in pulmonary embolism (4-Level Pulmonary Embolism Clinical Probability Score and YEARS) [18,19], the DAYS score was developed to enhance the number of patients deferred for further workup by increasing the D-dimer threshold while retaining sufficient safety [11]. The study identified DVT being the most likely diagnosis as one of the most predictive items. The item was inversely scored to the alternative diagnosis being more likely category in the Wells score. Although a reasonable methodological approach, it is uncertain whether the absence of an alternative diagnosis can be translated into that DVT is the most likely diagnosis. Moreover, the implicitness of this item may indicate reproducibility issues.

The prevalence of DVT in this study was 20%, slightly higher than the overall prevalence reported in the DAYS study (17%) [11]. This discrepancy could potentially explain the increased number of false negatives in the DAYS score. However, a more plausible explanation is the wide variation in DVT prevalence, which ranged from 6% to 39% among the individual studies from which the patients in DAYS study were derived [[20], [21], [22], [23]]. However, the proportion of patients classified as having DVT unlikely in this cohort is similar to the original study (20% vs 27%). Notably, the prevalence of DVT in patients classified as DVT unlikely was almost identical between the 2 studies (6.9% vs 6.8%), making it less likely that the reason for inadequate safety is the higher prevalence of DVT in our cohort, as most missed DVT cases were in the DVT unlikely group.

Most of the missed DVT cases by the DAYS score were distally located (*n* = 7) and occurred in the group classified unlikely with D-dimer between 0.5 μg/mL and 1.0 μg/mL. Several potential explanations exist for this finding. Two of the included studies in the DAYS study evaluated the patients only with proximal CUS [20,23]. In contrast, 33% of the patients in this study had a distal DVT, as all patients were evaluated with whole-leg CUS [13]. Additionally, distal DVTs are generally smaller in size, which may result in less frequent abnormal D-dimer [24]. Importantly, it has been argued that distal DVTs, particularly those located distal to the trifurcation of the popliteal vein, may not require anticoagulant treatment as it does not affect the 3-month incidence of VTE [25]. This statement was reinforced by a recent clinical management study, which only performed proximal CUS and reported no DVT events at follow-up in patients with low clinical probability and a D-dimer of <1.0 μg/mL [26]. Although speculative, if the 7 distally located DVTs in this study were not considered false negatives, the DAYS score would still not meet the overall safety threshold as the failure rate was 0.9% with the upper limit of the 95% CI reaching 2.8.

Notably, the failure rate defined in the DAYS score study is more stringent compared with current and historical literature, which suggests an average safety threshold of <2% or <4.4% when considering the upper bound of the CI [3,4,27]. We were unable to find any literature supporting the notion that the failure rate should not exceed the upper bound of the CI at 2%. If adequate safety would have been defined according to previous thresholds (ie, 4.4%), the DAYS score would have met the safety margin and been regarded as externally validated.

The DAYS score was able to identify an additional 14% patients in whom further diagnostic workup would not be required, compelling results, albeit, in this study, at the cost of safety. The new DVT score developed by our group demonstrated modestly improved safety and performance, as expected, compared with those of the DAYS score, given that it was tested on the same sample it was derived from.

The performance of the DAYS score and the new score highlight the challenge of balancing the need to ensure safety while reducing the use of diagnostic imaging. Although safety thresholds have been established for clinical decision rules in VTE, there is a lack of clear guidance in the literature regarding acceptable levels of efficiency.

The strengths of this study are the utilization of prospectively collected data and the availability of the original Wells score for all patients. Furthermore, the 20% prevalence of DVT ensures testing of the decision rules in a representative population, contributing to reliable diagnostic performance results. Finally, all DVT diagnoses were confirmed by ultrasound.

However, this study has some limitations that warrant mentioning. While the data used to test the decision rules were collected prospectively, this study was retrospective, making it liable to bias. Consequently, the subjective item of the DAYS score may have performed suboptimally, particularly since it required an inverse scoring. However, this study adhered to identical methodology used in the original study to mitigate this potential issue. A prospective management study, with proximal DVTs as the primary outcome, would probably offer the optimal method for thorough validation of the DAYS score by comparing it with an established diagnostic algorithm such as the simplified Wells score for DVT. In addition, as we could not distinguish between acute and chronic DVT in cases with DVT recurrence in the ipsilateral leg, some cases might have been incorrectly regarded as acute DVT when in fact they were chronic. Finally, distally located DVTs accounted for 33% of all confirmed DVTs, and due to the nature of the data, we were unable to differentiate between the types of distal DVTs. Given the ongoing debate regarding the necessity of anticoagulant treatment for distal DVTs, including only proximal DVTs as an outcome may have been more appropriate. However, even when considering only the proximally located missed DVTs, the DAYS score still failed to meet the predefined safety threshold. Unfortunately, data on ethnicity were not recorded. Given that most patients were Norwegian, the findings might have limited generalizability to populations comprising ethnic groups other than Caucasians.

## 5 Conclusion

The DAYS score did not meet the predefined criteria of safety in this external validation study. Although compelling to increase the D-dimer threshold, as has been the case with pulmonary embolism, further studies are needed to ensure the safety of such decision rules in patients with suspected DVT.
